# Cracking the Code of Digital Discomfort Through the Dynamic Fusion of Matrix Rhythm Therapy and Physiotherapy Exercises for Text Neck Syndrome

**DOI:** 10.7759/cureus.58085

**Published:** 2024-04-11

**Authors:** Divya Gohil, Reena S Kathed, Tushar J Palekar

**Affiliations:** 1 Department of Kinesiology and Movement Sciences, Dr. D. Y. Patil College of Physiotherapy, Dr. D. Y. Patil Vidyapeeth, Pune, IND; 2 Department of Orthopedics, Dr. D. Y. Patil College of Physiotherapy, Dr. D. Y. Patil Vidyapeeth, Pune, IND

**Keywords:** text neck syndrome, physical therapy, neck pain, mobile addiction, matrix rhythm therapy, case report

## Abstract

Text neck syndrome refers to the excessive use of electronic devices such as laptops, mobile phones, and so on, which causes prolonged and continued forward bending of the neck, leading to a strain in the muscle, causing muscle imbalance, and leading to poor posture. In this article, we focus on a case of a 22-year-old female who has a daily average screen time of around four to five hours, which leads to stress on cervical muscles that further develop into tightness and cause poor posture. She is managed with physiotherapy treatment that focuses on reducing pain and increasing the strength of the individual. The physiotherapy treatment focuses on the prevention of further damage to the cervical muscles and educating the individual to perform minimum forward bending by providing ergonomic advice, reducing pain, and improving range of motion.

## Introduction

In this tech-driven era, text neck syndrome has emerged as a prevalent musculoskeletal ailment, marked by persistent neck pain and dysfunction resulting from the prolonged use of electronic devices [1]. This condition arises from the emergence of cervical spinal degeneration because of the repetitive stress induced by prolonged forward head flexion [2]. Around 75% of the world's population is thought to dedicate significant daily hours to using handheld devices with their heads in a forward-flexed position [3]. The human head weighs about 10-12 pounds in a neutral position. As the head tilts forward while looking at the screen, the weight on the neck muscles is effectively increased [4,5]. This case study investigates how the integration of matrix rhythm therapy with conventional physiotherapy exercises proves effective in the management of text neck syndrome.

## Case presentation

**Figure 1:**
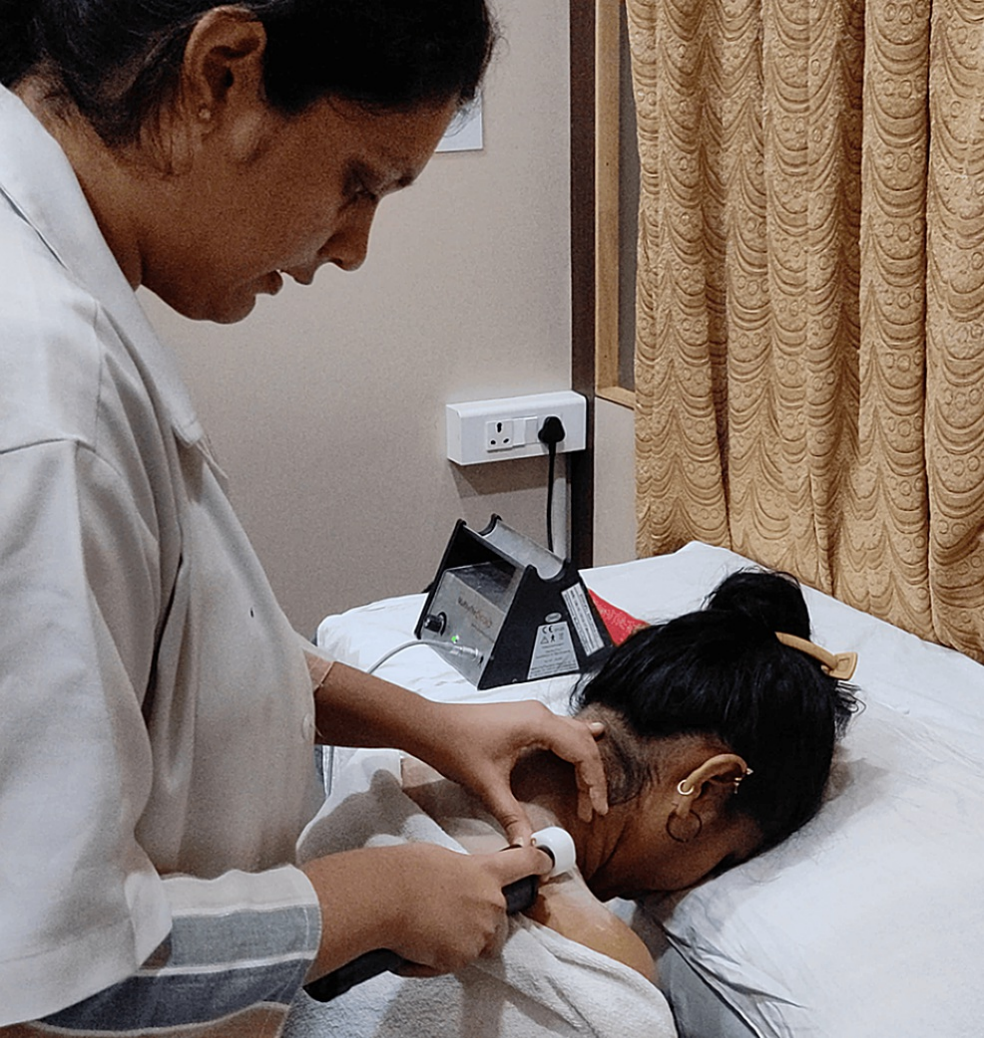
Matrix rhythm therapy technique

A 22-year-old female, currently pursuing a medical degree, has been experiencing neck pain and significant stiffness over the past three months. The pain, which increases during neck movements, has been identified as intermittent. Another contributing factor to her discomfort is the daily hunching of the neck over using her computer and smartphone. The patient agrees to the experience of dizziness or headaches. However, she highlights that her pain intensifies during prolonged periods of phone usage, coupled with forward bending of the neck, for around four to five hours daily. Moreover, she noticed the restriction in her neck movements; therefore, she consulted a doctor where normal medications for neck pain were advised with rest. However, her complaint was exacerbated by prolonged computer and smartphone use, totaling four to five hours daily. An MRI and an X-ray were not pursued. Pain intensity was 7/10 on activity, and, when on rest, it was 5/10, which she described as an aching, with the discomfort intensified on palpation of the trapezius muscle; moreover, grade 3 tenderness was revealed according to the tenderness grading scheme characterized by severe sensitivity carried by withdrawal. The palpation of the muscle led to diagnosing the trigger point on the trapezius muscle as it was tender, tense, and felt similar to a taut band where the pain traveled toward the head and shoulder after palpating it. The palpation was done using the two techniques through pincer and flat palpation, which resulted in the diagnosis of a taut band. Forward head posture and asymmetrical shoulders were observed before giving the treatment. Reduced cervical spine movements and muscle weakness were noted. The initial Neck Disability Index score was 46% based on the Neck Disability Index scale. The below figure shows the matrix rhythm therapy (MaRyThe©, Gröbenzell, Germany) equipment used for the treatment (Figure 1).

Timeline of the current episode

On August 10, the patient started experiencing neck pain, and it increased in intensity, leading her to visit the doctor. The doctor prescribed medications (painkillers), and she was sent back home and advised to rest. On December 15, the patient visited a physiotherapy clinic as her pain did not subside continuously. At this point, the range of motion and strength of the patient was reduced. Throughout physiotherapy treatment, there was an improvement in pain and range of motion including strength and mobile usage.

Diagnosis

Differential diagnoses include vertigo, cervical radiculopathy, tension headache, stress-related issues, and muscle strain. The assessment revealed a reduced craniovertebral angle causing forward head posture, likely because of text neck syndrome from excessive electronic device use. Cervical muscle stiffness also supports this diagnosis. 

Following a comprehensive assessment focusing on pain, range of motion, and mobile usage, text neck syndrome was diagnosed, ruling out other potential causes because of the absence of accompanying symptoms such as dizziness, radiating pain, or tingling numbness. Ergonomic modifications and posture corrections were implemented post-diagnosis, resulting in notable improvements in pain relief, range of motion, and overall strength.

Physiotherapy intervention

**Figure 2:**
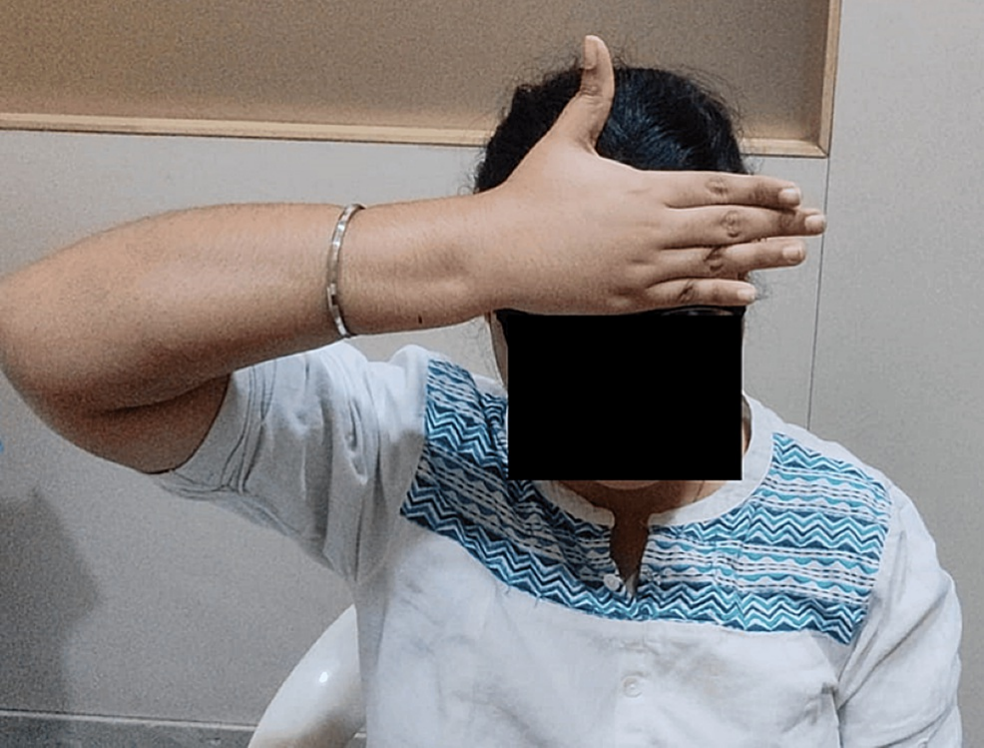
Subject performing neck isometric exercises

From December 15th-22nd, a seven-day physiotherapy intervention focused on pain reduction and stiffness alleviation initially, employing techniques such as matrix rhythm therapy, manual therapy, and stretching. Range of motion exercises and neck isometrics were followed for stiffness reduction. Strengthening exercises were introduced from day four to enhance cervical muscle stability. The long-term goals aimed at sustaining pain relief, improving range of motion, increasing muscle strength, and addressing excessive phone usage for posture correction to enhance quality of life (Tables 1-6) (Figure 2).

**Table 1:** The range of motion assessed on day one of rehabilitation

Pre-rehabilitation: Day 1				
	Active		Passive	
Movement	Right	Left	Right	Left
Cervical flexion	20° (0-60°)		28°	
Cervical extension	30° (0-70°)		36°	
Cervical rotation	25° (0-75°)	30°	28°	32°
Cervical side flexion	30° (0-45°)	35°	33°	37°

**Table 2:** The range of motion for day seven posttreatment

Post-rehabilitation: Day 7				
	Active		Passive	
Movement	Right	Left	Right	Left
Cervical flexion	50° (0-60°)		55°	
Cervical extension	45° (0-75°)		50°	
Cervical rotation	40° (0-70°)	45°	42°	45°
Cervical side flexion	42° (0-45°)	45°	45°	46°

**Table 3:** The pre- and post-day one and day seven pain intensity

Vas	On rest	On movement
Pre	6/10	9/10
Post	2/10	4/10

**Table 4:** The pre- and post-neck disability of the individual

Neck Disability Index	Pre	Post
	52%	30%

**Table 5:** The CV angle assessed pre- and post-days one and seven, respectively CV: craniovertebral

CV Angle	Pre	Post
	44.29	46.10

**Table 6:** Physiotherapy intervention from day one to day seven

Treatment plan	Exercise protocol	Duration and frequency	Rationale
Patient education	To educate about the condition and regarding the importance of exercise and reduction of screen time	At the initial phase of starting the treatment when the patient visits	To make the patient actively involve and understand the precautions
Pain reduction (Days 1–3)	Heat therapy-hot pack	10 minutes twice a day	To promote relaxation of muscle, decrease the stiffness, and improve healing
To prevent loss of strength (Days 1–3)	Neck isometric exercises in every direction: forward, backward, sideways, rotation	10 repetitions twice daily	To improve physical endurance and posture by maintaining the strength and maintaining range of motion
To increase the strength (Days 3–7)	Neck exercises in every direction with the use of TheraBand: forward, backward, sideways, rotation	10 repetitions once daily	To improve and maintain the strength
Posture correction exercises (Days 3–7)	Shoulder and neck muscle stabilization exercises: chin tucks, shoulder shrugs, scapular retractions. Introduction of stretching regime for suboccipital and trapezius muscle.	10 repetitions twice daily	To minimize the strain on the muscles that cause imbalance
Matrix rhythm therapy (Days 1, 3, 5, 7)	Matrix therapy over the trapezius and splenius capitis muscles as they get tensed while using the mobile phone or screen.	30 minutes per session	To reduce the stiffness by increasing the adenosine triphosphate synthesis and regeneration of cell activity

## Discussion

Matrix rhythm therapy, being the advanced treatment formula to reduce stiffness and increase range of motion, diminishes pain by working on the cells through the body’s physiological rhythmic oscillations. This frequency aligns with the body to restore disrupted rhythms at the cellular level. The therapy enhances oxygen supply by improving microcirculation, leading to better energy production. As a result, an immediate effect is seen on tissues, muscles, and fascia. This relaxation lasts longer as the therapy regulates cellular metabolic processes and enhances oxygen delivery to cells [6]. In this case report, the patient, when she came to us was in immense pain and provided a high Neck Disability Index score with a restricted range of motion. Immediately after the physiotherapy session, the patient felt relaxed, and the pain intensity reduced. The treatment plan spanned over seven days, incorporating a combination of exercise therapy and matrix rhythm therapy. The patient was able to get back to her daily tasks after the physiotherapy intervention provided to her. She was able to concentrate and work for more than her usual hours. Overall, the case study provided evidence for proper and successful physical therapy evaluation, examination, treatment, and outcomes. The patient was treated in the subacute phase, which made the prognosis excellent for the patient. Functional and objective assessments conducted at the beginning and conclusion of the treatment revealed significant improvements. The comprehensive approach, involving progressive range of motion, strengthening exercises, matrix rhythm therapy, postural education, retraining, and deep neck muscle retraining, proved to be an effective care plan for individuals in the acute/subacute stage.

In considering potential diagnoses, differential considerations encompassed conditions such as cervical radiculopathy, cervical spondylosis, and tension headaches, all of which exhibit symptoms that overlap with text neck syndrome. However, thorough evaluation excluded these alternatives as the patient did not present accompanying symptoms such as radiating pain, tingling numbness, or any tension headaches typically associated with these conditions.

## Conclusions

Matrix rhythm therapy, combined with traditional physical therapy, played a crucial role in expanding the joint range of motion, supporting skeletal muscle repair, and facilitating the regeneration of injured muscles. The therapy improved the patient’s well-being and enhanced quality of life. This physiotherapy technique, when combined with conventional physiotherapy, not only benefited individuals with pain relief and other physical limitations but also regained strength, flexibility, and confidence in performing day-to-day activities with ease.
